# Association between entomological indices and malaria test positivity rates in Western Kenya: implications for surveillance

**DOI:** 10.1186/s12936-026-06073-x

**Published:** 2026-08-08

**Authors:** Brenda Omala, David Mburu, Maurice Ombok, Vincent Moshi, John Gimnig, Nicole L. Achee, John P. Grieco, Bernard Abong’o, Eric Ochomo

**Affiliations:** 1 Pwani University Bioscience Research Centre (PUBReC), Pwani University, Kilifi, Kenya; 2 Center for Global Health Research, Kenya Medical Research Institute, Kisumu, Kenya; 3 Division of Parasitic Diseases and Malaria, Centre for Global Health, Centre for Disease Control and Prevention, Atlanta, GA 30333 USA; 4 Department of Biological Sciences, Eck Institute for Global Health, University of Notre Dame, Notre Dame, USA; 5 Vector Group, Liverpool School of Tropical Medicine, Liverpool, UK

## Abstract

The relationship between malaria entomological indices and epidemiological outcomes remains poorly understood at local scales in western Kenya, limiting evidence-based surveillance strategies. This study evaluated associations between malaria test positivity rates and vector densities, human biting rates, and entomological inoculation rates (EIR) in a high-transmission setting. The study was conducted in five villages within Teso South sub-County, Busia County, Kenya, from March to June 2021. Village-level entomological indices including vector densities, human biting rates (HBR), and entomological inoculation rates (EIR) were calculated for *Anopheles funestus* and *An. gambiae s.l*. Concurrent malaria test positivity rates were obtained from outpatient department registers in the health facilities serving the five villages (n = 2377 cases). The relationship between entomological indices and malaria test positivity was analyzed using multilevel logistic regression models, accounting for individual- and village-level factors. During this period 1950 female anopheline mosquitoes, predominantly *An. gambiae s.l* (89.0%) and *An. funestus* (11.0%) were collected. Malaria test positivity rates varied significantly across villages (range: 23.6%-68.7%). Multilevel analysis revealed significant associations between malaria test positivity and both anopheline mean density (OR 1.11, 95% CI 1.08–1.15, *p* < 0.0001) and HBR (OR 1.35, 95% CI 1.17–1.57, *p* < 0.0001). However, an association between EIR and malaria test positivity was not observed (OR 1.04, 95% CI 0.96–1.13, *p* = 0.316). These findings suggest that one solution to address foci with higher malaria cases is to target transmission through vector control based on health facility surveillance. This information could be valuable for national malaria control programs in optimizing surveillance strategies and evaluating intervention effectiveness.

## Background

Despite significant advances in control measures, malaria remains a critical global public health challenge, particularly in sub-Saharan Africa. In the most recent World Malaria Report, there were an estimated 282 million malaria cases in 2024 with approximately 610,000 deaths globally [1]. In Kenya, while national prevalence was 5.6% according to the 2020 Kenya Malaria Indicator Survey, substantial regional variations persist, with Busia County reporting a prevalence of 38% [2]. Effective malaria control requires robust surveillance systems that can accurately monitor both entomological and epidemiological indicators [3]. However, these two aspects of surveillance are often conducted independently, creating potential gaps in our understanding of transmission dynamics. While epidemiological surveillance through health facility data provides valuable information about disease burden, it often suffers from reporting inconsistencies and coverage limitations. Entomological surveillance, though more resource-intensive, can provide early warnings of transmission risk and intervention effectiveness and can identify failure of vector control interventions due to the quality of implementation, insecticide resistance, and behavioral changes in the mosquito vector [4].

The World Health Organization's Global Vector Control Response (2017–2030) emphasizes surveillance as a crucial pillar for malaria elimination efforts [5]. National malaria control programs (NMCPs) conduct parallel but separate tracking of entomological and epidemiological indicators. These form the basis for evaluation of control interventions, monitoring temporal and spatial heterogeneity of *Plasmodium* parasitemia as well as malaria vectors [6]. Epidemiological data is often routinely collected passively through health facility records complemented with Malaria Indicator Surveys conducted once every 5 years. Entomological surveillance is often conducted irregularly, at relatively small spatial scales, and primarily for research or targeted surveillance purposes, limiting its impact on making informed decisions about wide administrative areas [7, 8]. This separation raises important questions about the relationship between these indicators and whether one set could effectively predict the other, potentially optimizing resource allocation in surveillance programs.

Since the entomological and epidemiological datasets are usually collected by independent groups at the NMCPs, efforts to understand their relatedness are rare. For new vector control paradigms, WHO requires evidence of epidemiological efficacy in at least two countries [9], with less emphasis on entomological outcomes. Entomological surveillance is currently less systematically utilized but opportunities exist to increase coverage, which is desirable if there are clear relationships between entomological surveillance data and clinical outcomes, as this paper seeks to evaluate.

While previous studies have examined relationships between individual indices and transmission [10, 11], few have comprehensively analyzed multiple entomological indicators simultaneously with epidemiological outcomes. Key knowledge gaps include the predictive value of multiple entomological indices in combination and the spatial heterogeneity of these relationships at village level. This study investigated the relationship between key malaria entomological indices (vector densities, entomological inoculation rates, and human biting rates) and malaria test positivity rates in five villages within Busia County, western Kenya. We hypothesized that entomological indices would show significant associations with malaria test positivity rates, potentially serving as predictive indicators for transmission intensity. Our findings could inform more efficient and integrated approaches to malaria surveillance and response, particularly in resource-limited settings.

## Materials and methods

### Study design and study population

**Fig. 1:**
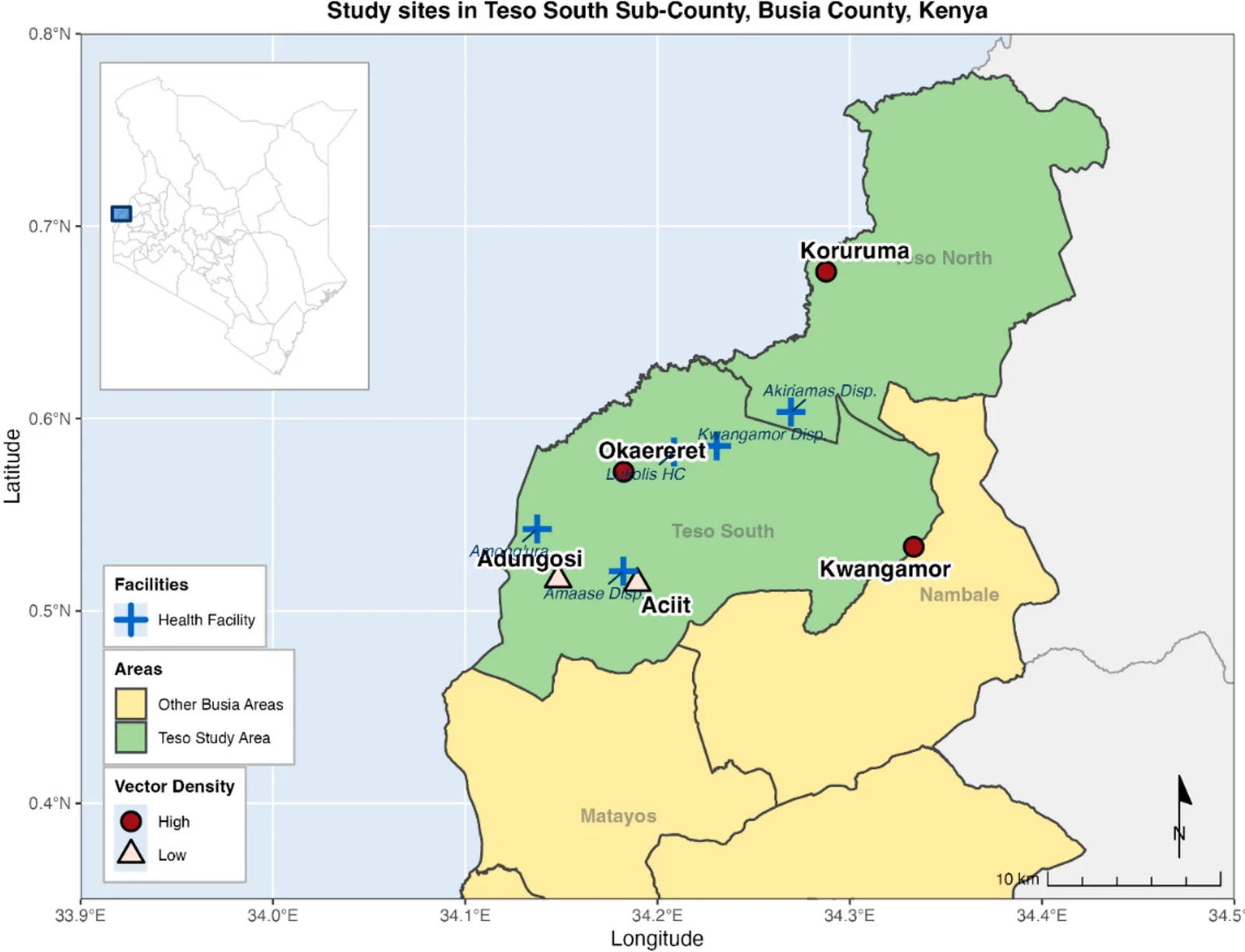
A map showing study sites in Teso South sub counties

This study was nested within a cluster randomized control trial (cRCT) evaluating the epidemiological impact of spatial repellent’s impact on malaria incidence in western Kenya [12]. The study was carried out between March and June 2021, corresponding to the long rainy season, in five villages within Teso South sub-County, Busia County: Adungosi, Aciit, Kwangamor, Koruruma, and Okaereret (Fig. 1). These villages were selected based on completeness of health facility data. Epidemiological data were obtained from the health facility records in the specific villages where mosquito collection was performed for correlation purposes. The study was conducted during the baseline period prior to the deployment of spatial repellents.

The study area is characterized by moderate to high rainfall (760–2000 mm annually) distributed across two seasons: long rains (March–May) and short rains (October–November). Busia County, situated within the Lake Victoria region, reports the highest malaria prevalence (38.5%) in Kenya. The region's primary economic activities include subsistence farming and cash crop cultivation (sugarcane and cassava). Insecticide-treated nets (ITNs) are the primary vector control intervention in the area.

### Eligibility criteria

We included all cases, from March 1 to June 30, 2021, that were from the five study villages and with complete variables of interest including patient’s identity number, test results, sex, reporting month and village of residence. Cases that failed to meet any of the mentioned parameters were excluded in the analysis.

### Sample size and sampling strategy

Sample size determination followed the protocol of the parent spatial repellent trial. For entomological surveillance, we randomly selected 10 households per village per month (total n = 198 sampling events) using a computerized random number generator. For epidemiological data, we analyzed all available malaria test records from the five health facilities serving these villages during the study period (n = 2377 records).

### Health facility surveillance

Outpatient department data (OPD) from five health facilities adjacent (within 5 km) to the study villages were retrieved and used to extract records of individuals coming from the study villages. Five facilities; Akiriamas Dispensary, Amaase Dispensary, Among’ura Dispensary, Kwangamor Dispensary and Lukolis Model Health Center (referral facility), participated in this study. Data was abstracted from Ministry of Health (MoH) OPD under five (204 A) and over five (204 B) registers of the chosen hospitals for similar months as of entomological data collection. A smart phone camera was used to photograph every page of the registers, and the images were converted into portable document formats (PDFs. Patients’ confidentiality was ensured by covering the column containing their names and other personal identifiers when taking photographs. The information was then recorded in Microsoft Excel.

### Outcome measures from the health registers

The variables of interest for the study included: name of health facility (HF), age, sex, month, malaria test results regardless of parasite species. Malaria test positivity rate was determined as the fraction of persons diagnosed with malaria, over the total number of individuals tested for malaria at the health facility for each study village. The tests were conducted by malaria rapid diagnostic tests (mRDT). To facilitate linkage of the HF data to the respective village, patients’ area of residence was also abstracted as recorded on the OPD registers, and the outcome measures were calculated per village.

### Vector surveillance

Mosquito collection was conducted using CDC light traps indoors in five villages between March and June 2021. Data from the indoor placed traps were used to estimate human biting rates (HBR) [13]. Ten (10) households were randomly selected in every village each month and used as study units for mosquito sample collection. Other metadata collected alongside the entomology questionnaire included: whether eaves were open or closed, type of roof and wall, information on cooking in the rooms as well as utilization of other mosquito control methods for instance mosquito coils, the night before collection. The traps were installed in sleeping rooms next to an occupied bednet, one (1) meter height at 1800 h in the evening, left to run all night and collected at 0700 the following morning. Trapped mosquitoes were then moved to the site laboratory in trap cups for morphological characterization and further analyses.

### Identification of *Anopheles* mosquito species and sporozoites detection

Mosquito samples collected from the selected villages were identified morphologically using the taxonomical keys of Coetzee [14]. Legs, wings and abdomen were cut from the mosquitoes to be utilized in polymerase chain reaction (PCR) examination [15] for species level identification of members of *An. gambiae* Complex and *An. funestus s.l* group. Molecular identification was done on up to a hundred samples per village per month. Sporozoite infection was detected using a sandwich enzyme linked immunosorbent assay (ELISA) as described by prior studies [16] on the heads and thoraces of the same mosquitoes per village used for PCR species identification.

### Data analysis

The analytical approach focused on examining the relationship between entomological indices and malaria test positivity rate while accounting for both individual and village-level variations. All analyses were conducted using STATA or SAS statistical software, employing a systematic multilevel modelling approach to handle the nested structure of the data. To prepare the data for analysis, the distribution properties were examined. The Shapiro–Wilk test revealed non-uniform distribution patterns in the key variables (*p* < 0.05), leading to the employment of non-parametric methods where appropriate. The Kruskal–Wallis test was used to examine differences in mosquito means between villages, as this test does not assume a normal distribution.

### Determining malaria vector mean densities

Vector abundance was assessed using descriptive statistics (means, proportions, and 95% CI). The quotient from malaria vectors collected and number of households where the mosquitoes were collected in a village was considered as malaria vector density. This indicator gives a general view of information on malaria vector density per house in a village.

### Determining sporozoites infectivity (SR)

Sporozoite rates were derived by the formula given below: -

$$SR=\frac{Total female anopheline mosquitoes positive for P.f sporozoites}{Total number of mosquitoes tested}$$

### Determining human biting rates

Direct calculation approach was made from female *Anopheles* mosquitoes collected using indoor CDC-LTs and the number of sleepers inside a mosquito net with an assumption that the mosquitoes were seeking the human host under the bed net next to which the light trap was hung [13]. In this study, indoor CDC light traps were used as an alternative approach to approximate HBR. Mosquito collections from CDC light traps placed near occupied sleeping spaces provide a proxy measure of indoor host-seeking mosquito density. This approach has been widely adopted as a safer and more operationally feasible alternative to HLC, particularly for routine surveillance, although it may require site-specific calibration to improve accuracy. The analysis was done per village using formula as shown below:

$$HBR=\frac{Total female anopheline mosquitoes collected by light trap}{Total number of sleepers in the collection structures}$$

### Determining entomological inoculation rate (EIR)

Entomological inoculation rates were calculated using the PCR sporozoite rate results combined with the overall number of mosquito captures to generate an estimated species-specific HBR. EIR was obtained by multiplying sporozoites rates, human biting rates using a time period (4 months/122 days) as guided by WHO and other studies [17, 18].The EIR was calculated as follows: -

$$EIR=Human biting rate \left(HBR\right)\times Sporozoite Rate \left(SR\right)\times 122 days(4 months)$$

### Association between entomological and epidemiological indicators

The main analysis utilized multilevel logistic regression models to account for the hierarchical nature of the data, where individuals (level 1) were nested within villages (level 2). Individual ages were centred around the grand mean to facilitate interpretation and reduce multicollinearity. For village-level measures, the researchers created non-self-aggregate values, excluding each individual's data point from their respective village-level calculations. The modelling process proceeded through four sequential stages: In the first stage, an empty model (null model) containing only the random intercept for villages was fitted. This calculation of the intraclass correlation coefficient (ICC) revealed that 14% of the variance in malaria test positivity was attributable to village-level differences, providing justification for the multilevel modelling approach. The second stage involved construction of constrained intermediate model incorporating all individual-level variables (age, sex) and village-level variables (vector densities, HBR, EIR). Due to the high collinearity between them, separate models were constructed for each village-level variable plus the individual level variables. These models elucidated the main effects of predictors while accounting for the nested data structure. In the third stage, the research team developed augmented intermediate models incorporating random slopes for individual-level variables. Comparison with the constrained models using likelihood ratio tests determined whether allowing individual-level variable effects to vary across villages improved model fit. The final stage involved fitting models with cross-level interactions between age and the entomological predictors (mean densities, HBR, and EIR). This analysis tested whether entomological indices' effects on malaria test positivity varied with individual age. For all models, odds ratios with 95% confidence intervals were calculated, considering P-values less than 0.05 as statistically significant. Model fit was assessed using deviance statistics, and residuals were examined for model assumption violations.

Sensitivity analyses were conducted through: (1) the removal of influential observations and reassessment of key relationships, (2) testing of alternative correlation structures and (3) examination of missing data impact on conclusions. All statistical tests employed a significance level of α = 0.05. Model assumptions were verified through residual plots and influence diagnostics. Result reporting included measures of uncertainty (confidence intervals) alongside point estimates to provide comprehensive understanding of finding precision.

This approach allowed for addressing the hierarchical data structure, control for potential confounding at both individual and village levels, examination of cross-level interactions, generation of robust estimates of relationships between entomological indices and malaria test positivity and quantification of estimate uncertainty. The analysis provided the foundation for understanding the relationship between entomological indices and malaria transmission patterns in the study area, while accounting for the complex nature of the data structure.

### Study reporting scope

The manuscript reported only confirmed malaria vector species within the *Anopheles gambiae* complex and *An. funestus*. Sporozoite rate had species specific reporting of the same complexes. Other non-vector species were excluded to maintain focus on transmission-relevant species in the study area.

### Ethical considerations

Ethical clearance to conduct the study was obtained from the Kenya Medical Research Institute – Scientific and Ethics Review Unit (KEMRI-SERU) number 3870 and Pwani University Institutional Scientific and Ethics Review Committee, approval number ISERC/MSc/048/2022. The study was approved by the US Centers for Disease Control Institutional Review Board (IRB# 7252) through a reliance agreement with KEMRI-SERU. During routine mosquito collection by CDC-LT verbal consents were obtained from household heads. All methods were performed according to stipulated guidelines and regulations. Permission for secondary data usage was obtained from facility in charge of respective health facilities following presentation of the ethical clearances from KEMRI-SERU and Pwani University.

## Results

### Malaria test positivity rates

The study findings were derived from a comprehensive analysis of both entomological and epidemiological data collected from five villages in Busia County, western Kenya. A total of 18,299 facility records were abstracted from the Ministry of Health (MOH) under five and over five years facility outpatient department registers. Of these 2377 malaria test records met the inclusion criteria for analysis. Of those who were tested for malaria, 61.8% (n = 1470) were females and 38.2% (n = 907) were males. The age distribution was: < 5 years: 25.3% (n = 601), 5–14 years: 35.7% (n = 849), 15–45 years: 28.4% (n = 675) and > 45 years: 10.6% (n = 252). Mean age was 16.8 years (SD: 15.4). The distribution of positive cases varied across the study villages, with the highest number of records being documented in Koruruma (n = 915), followed by Kwangamor (n = 690), while fewer cases were recorded in Okaereret (n = 211), Aciit (n = 311), and Adungosi (n = 250).

The test positivity rate was 52.4% among females and 57.5% among males. School going children aged between 5–14 years had the highest malaria test positivity rate TPR = 76.9%(95%CI 73.1–80.7%) while children under five years and individuals above 14 years in age had the lowest test positivity rates, TPR = 52.2% (95%CI 48.5–56.5%) and TPR = 45.4% (95%CI 42.4–48.3%) respectively. An overall malaria test positivity rate of 54.3% (95%CI 52.3–56.3%) was calculated across all study sites. Malaria test positivity rate varied across villages by age groups. Children aged 5–14 years consistently exhibited the highest positivity across all villages, ranging from 55.2% (95% CI 42.5–67.3) in Aciit to 89.8% (95% CI 79.9–95.2) in Okaereret. Among children aged < 5 years, positivity ranged from 17.1% (95% CI 10.2–27.3) in Adungosi to 63.0% (95% CI 57.0–68.6) in Kwangamor. In participants aged ≥ 15 years, positivity ranged from 22.7% (95% CI 16.7–30.0) in Adungosi to 68.7% (95% CI 62.8–74.0) in Kwangamor (Table 1). Significant heterogeneity in positivity rates was observed among villages, with the highest rate being recorded in Kwangamor (68.7%), followed by Okaereret (64.5%) and Koruruma (56.1%). Substantially lower rates were documented in Aciit (35.0%) and Adungosi (23.6%) (Table 2).

**Table 1:** Participant characteristics by age groups and villages (95%CI), TPR (test positivity rate)

Village	TPR, < 5 years	TPR,5–14 years	TPR, ≥ 15 years
Kwangamor	167/265, **63.0%** (57.2–68.8)	133/171, **77.8%** (71.6–84.0)	173/252, **68.7%** (63.0–74.4)
Koruruma	153/263, **58.2%** (52.2–64.2)	176/225, **78.2%** (72.8–83.6)	184/427, **43.1%** (38.4–47.8)
Okaereret	34/61, **55.7%** (43.2–68.2)	53/59, **89.8%** (82.1–97.5)	49/91, **53.8%** (43.6–64.0)
Aciit	29/97, **29.9%** (20.8–39.0)	32/58, **55.2%** (42.4–68.0)	48/155, **31.0%** (23.7–38.3)
Adungosi	13/76, **17.1%** (8.6–25.6)	12/21, **57.1%** (35.9–78.3)	34/150, **22.7%** (16.0–29.4)

The values shown in bold are the TPR for each age group in each study village

**Table 2:** Distribution of records and malaria cases in the study villages (95%CI)

Village	Total records	Malaria positive cases	Malaria test positivity rate (95% CI)
Kwangamor	690	474	68.7%(65.2–72.2)
Koruruma	915	513	56.1%(52.9–59.3)
Okaereret	211	136	64.5%(58.0–70.9)
Aciit	311	109	35.0%(29.8–40.4)
Adungosi	250	59	23.6%(18.3–28.9)
Total	2377	1291	54.3%(52.3–56.3)

Pairwise comparisons of the malaria test positivity rates between the study villages were conducted using Marascuilo Procedure (Table 3). The test showed significant difference between the village proportions apart from Aciit and Adungosi, Koruruma and Okaereret, Kwangamor and Okaereret.

**Table 3:** Marascuilo procedure for pair-wise comparison of malaria test positivity rates between study villages

Pair	Abs. diff	Critical. range	Abs. diff critical. range	Significant*
Aciit, Adungosi	0.114	0.117	−0.003	N
Koruruma, Okaereret	0.084	0.113	−0.029	N
Kwangamor, Okaereret	0.042	0.115	−0.073	N
Kwangamor, Koruruma	0.126	0.074	0.052	Y
Koruruma, Aciit	0.21	0.097	0.113	Y
Aciit, Okaereret	0.294	0.131	0.163	Y
Koruruma, Adungosi	0.325	0.097	0.228	Y
Kwangamor, Aciit	0.336	0.1	0.237	Y
Adungosi, Okaereret	0.409	0.131	0.278	Y
Kwangamor, Adungosi	0.451	0.099	0.352	Y

^*^‘N’ and “Y” indicate data of no statistical significance and significant difference, respectively

### Total mosquitoes collected

**Fig. 2:**
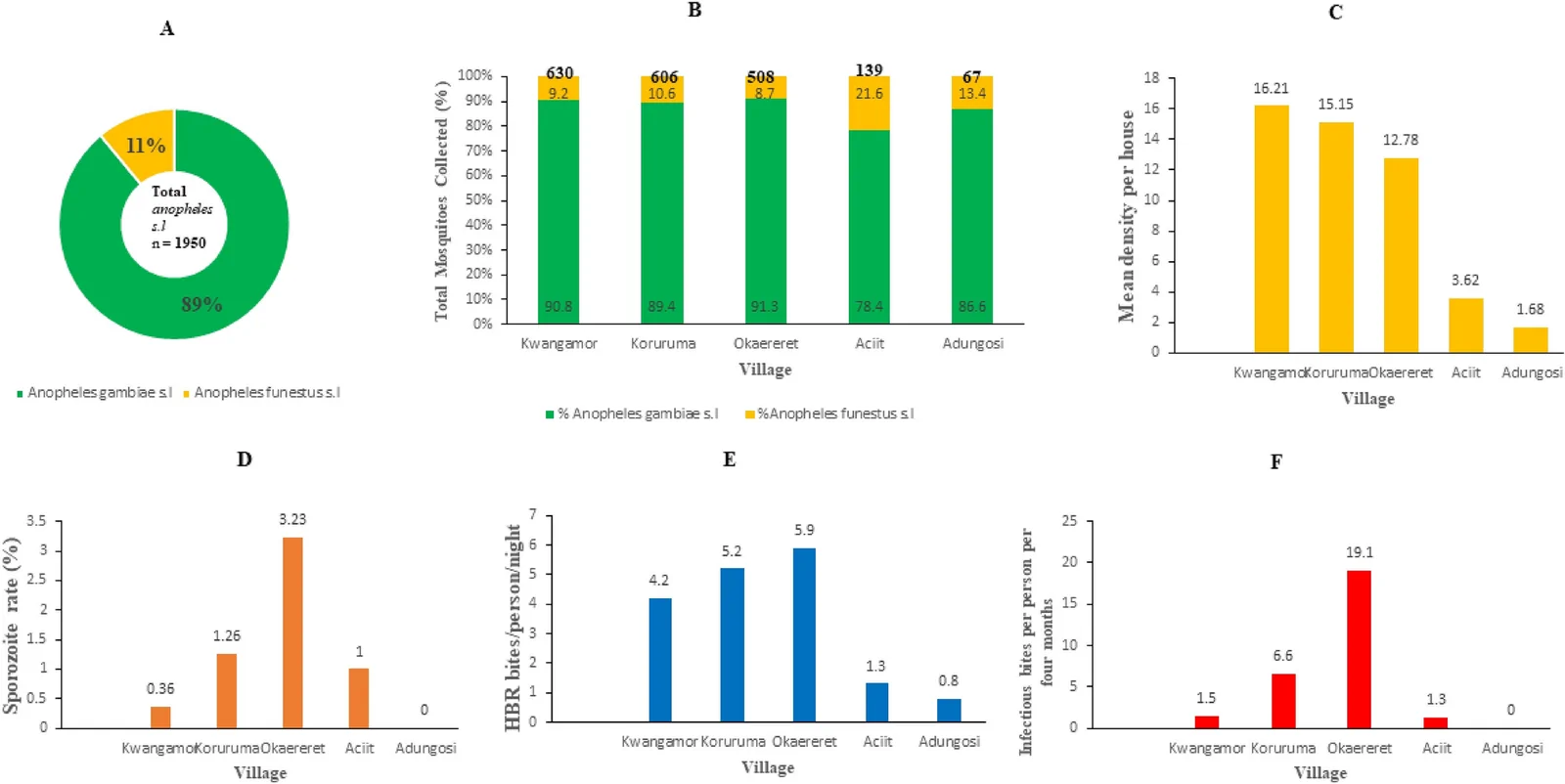
Entomological Indicators (**A** overall species composition, **B** complex species composition by village, **C** Mean density by village, **D** overall complex species sporozoite rate by village, **E** overall complex species HBR by village, **F** overall complex species EIR by village)

In the entomological surveillance, 1950 female anopheline mosquitoes were collected and identified. The predominant species was *An. gambiae s.l*., comprising 89.0% (n = 1745) of the total collection, followed by *An. funestus* at 11.0% (n = 205) (Table 4). A proportion of the *Anopheles* mosquitoes (n = 661) was also identified by PCR and *An.gambiae s.s* was the predominant member of the *An. gambiae* complex (78.2%) The overall vector density across the study area was calculated at 10.0 mosquitoes per house per night, although marked variations were observed between villages. The highest densities were recorded in Kwangamor, Koruruma, and Okaereret, where 16.21 (95% CI 14.02–18.40), 15.15 (95% CI 13.10–17.20), and 12.78 (95% CI 10.89–14.67) mosquitoes per house per night were observed respectively. Lower densities were observed in Aciit and Adungosi, where only 3.62 (95% CI 2.28–4.42) and 1.68 (95% CI 1.10–2.26) mosquitoes per house per night were recorded (Fig. 2).

**Table 4:** Sporozoite rates by mosquito species with the pairwise statistical comparison table

Species	Positive/examined	Sporozoite rate % (95% CI)	Comparison	OR (95% CI)	*p-*value
*An.* *funestus* s.s	5/89	5.6 (2.4–12.5)	vs *An.* *gambiae* s.s	4.94 (1.47–16.56)	0.029
*An.* *gambiae* s.s	6/505	1.2 (0.5–2.6)	vs *An.* *arabiensis*	0.59 (0.07–5.09)	1.000
*An.* *arabiensis*	1/50	2.0 (0.4–10.5)	vs *An.* *funestus* s.s	2.91 (0.33–25.73)	0.670

^*^Values are presented as sporozoite-positive mosquitoes/total examined, with Wilson 95% confidence intervals. Pairwise comparisons were performed using two-sided Fisher's exact tests. OR = odds ratio

### Human biting rates

The human biting rate (HBR) was determined across the study area, with an estimated 2.95 mosquitoes per person per night. The HBR was also calculated for the complex species. *Anopheles funestus s.l* exhibited the highest (8.7-fold) observed HBR 0.3 bites per person per night while *Anopheles gambiae s.l* had 2.6 bites per person per night. Variation in HBR was noted among the villages, with the highest rates being observed in Okaereret (5.9 mosquitoes/person/night) (95% CI 5.05–6.75), Koruruma 5.2, (95% CI 4.40–6.00), and Kwangamor, 4.2, (95% CI 3.45–4.95). In contrast, lower rates were documented in Aciit 1.3, (95% CI 0.85–71.75) and Adungosi 0.8, (95% CI 0.44–1.16). These variations were statistically significant (*p* < 0.001) (Fig. 2).

### Sporozoite rates

A total of 644 samples were tested for species specific sporozoite infectivity rate. The overall mean positivity rate was 1.71%. Sporozoite infection was detected in all three anopheles species. *Anopheles funestus s.s.* exhibited the highest observed sporozoite rate (5/89; 5.6%, 95% CI 2.4–12.5%), although this estimate was based on fewer mosquitoes tested than *An. gambiae s.s.* It was followed by *Anopheles arabiensis* (1/50; 2.0%, 95% CI 0.4–10.5%) and *Anopheles gambiae s.s.* (6/505; 1.2%, 95% CI 0.5–2.6%) (Table 4). Pairwise Fisher's exact tests showed that the sporozoite rate was significantly higher in *An. funestus s.s.* than in *An. gambiae s.s.* (OR = 4.94, 95% CI 1.47–16.56; P = 0.029). No significant differences were observed between *An. funestus s.s.* and *An. arabiensis* (OR = 2.91, 95% CI 0.33–25.73; P = 0.67) or between *An. gambiae s.s.* and *An. arabiensis* (OR = 0.59, 95% CI 0.07–5.09; P = 1.00) (Table 4). Village level sporozoite rates ranged from 0% (95% CI 0.00–0.00) in Adungosi to 3.23% (95% CI 2.15–4.31) in Okaereret; Kwangamor 0.36% (95% CI 0.05–0.67), Koruruma 1.26% (95% CI 0.62–1.90) and Aciit 1.00%, (95% CI 0.44–1.56) (Fig. 2).

### Entomological inoculation rates (EIR)

The entomological inoculation rate (EIR) was calculated for the study period, with an overall rate of 5.8 infectious bites per person. When analyzed by species, *An. gambiae s.s.* contributed 1.1 infectious bites per person, while *An. funestus* s.s. accounted for 0.81 infectious bites per person for four months. Analysis at complex level showed that *Anopheles gambiae s.l* had the highest EIR compared to *An. funestus s.l*, 4.2 versus 1.7 infective bites/person during the study period respectively. EIR varied across villages, with the highest rate recorded in Okaereret (19.1 infectious bites/person), (95% CI 15.1–23.1), followed by Koruruma 6.6, (95% CI 4.4–8.8). Lower rates were observed in Kwangamor 1.5, (95% CI 0.7–2.3) and Aciit 1.3, (95% CI 0.6–2.0), while no infectious bites were recorded in Adungosi 0.0, (95% CI 0.0–0.0) (Fig. 2).

### Relationship between malaria test positivity rates and age, anopheline mean densities, HBR and EIR

A multilevel logistic modeling approach of village- and individual-level factors as predictors of malaria test positivity was used. The null model showed an intraclass-correlation coefficient (ICC) of 0.14, suggesting that 14% of the variance in positive malaria test is accounted for by factors at the village level (between-village differences). Significant associations were identified between two entomological indices and malaria test positivity in three separate models. A positive association was established between vector density and malaria positivity, with an odds ratio of 1.11 (95% CI 1.08–1.15, *p* < 0.0001). Similarly, a positive association was determined between human biting rate and malaria positivity (OR: 1.35, 95% CI 1.17–1.57, *p* < 0.0001). Individuals had a 11%, and 35% increase in being infected with malaria when mean density of mosquito and human biting rate increases by one unit respectively. However, no significant relationship was observed between EIR and malaria test positivity (OR: 1.04, 95% CI 0.96–1.13, *p* = 0.316). In all models, children aged 5–14 years were significantly more likely to test positive compared to persons aged 15 years or older. In pairwise comparisons (data not shown), children aged 5–14 years were more likely to test positive compared to children under 5 years. There were no differences in the positivity rates between children under 5 years or persons 15 years and older. No significant associations were observed between sex and malaria positivity in any of the models. For the cross-level interactions, the effect of entomological factors on malaria test positivity did not differ significantly with individual age. Primary outputs from the models are provided in Table 5.

**Table 5:** Relationship between malaria test positivity rates and *Anopheles* mean densities, HBR, age, EIR and sex

Variable	OR (95% CI)	*p-*value
Model 1		
Sex (ref = male)	1.01 (0.84–1.21)	0.921
Age < 5 years (ref = ≥ 15 years)	1.18 (0.964–1.43)	0.111
Age 5–14 years (ref = ≥ 15 years)	3.39 (2.66–4.32)	< 0.001
Anopheline mean density	1.11 (1.08–1.15)	< 0.001
Model 2		
Sex (ref = male)	1.01 (0.84–1.21)	0.946
Age < 5 years (ref = ≥ 15 years)	1.18 (0.97–1.44)	0.109
Age 5–14 years (ref = ≥ 15 years)	3.38 (2.65–4.31)	< 0.001
Human biting rate (HBR)	1.35 (1.17–1.57)	< 0.001
Model 3		
Sex (ref = male)	1.01 (0.84–1.21)	0.927
Age < 5 years (ref = ≥ 15 years)	1.17 (0.96–1.43)	0.116
Age 5–14 years (ref = ≥ 15 years)	3.38 (2.66–4.31)	< 0.001
EIR	1.04 (0.96–1.13)	0.316

## Discussion

This study demonstrated strong associations between two entomological indices and malaria test positivity rates in western Kenya, providing critical evidence for optimizing surveillance strategies in high-transmission settings. The significant associations between vector density and human biting rate with malaria test positivity align with findings from similar high-transmission areas. Recent studies in Ethiopia [10], Tanzania [19], São Tomé and Príncipe [20] have similarly documented strong correlations between vector abundance and disease transmission, attributable to increased human-vector contact probability in areas with higher mosquito densities [11]. The particularly strong association with HBR reflects the predominance of highly anthropophilic *An. gambiae* s.s. and *An. funestus* in the study area [21], consistent with findings from other East African endemic regions where HBR provides direct measurement of human exposure to vector bites [22] [23].

The lack of significant association between EIR and malaria positivity contrasts with some previous studies [24] but may be explained by the uncertainty introduced by combining measurement error from vector densities as well as sporozoite rates. In small sample sizes, a few positive mosquitoes can substantially affect estimates of EIR. Other potential factors include ongoing vector control interventions, particularly widespread ITN use, may have reduced sporozoite rates as demonstrated in western Kenya intervention studies [25]. Additionally, the four-month study duration may have limited ability to capture the full EIR-transmission relationship [26], while complex immunity development in endemic areas can influence the relationship between infectious bites and clinical malaria [27].

The significant spatial heterogeneity in both entomological indices and malaria test positivity across villages aligns with recent comprehensive analyses of malaria transmission patterns in western Kenya [28, 29], where substantial micro-geographic variation has been documented within similar ecological zones [30, 31]. The observed heterogeneity can be attributed to local environmental characteristics, including altitude and land use patterns [31], variations in vector control coverage and effectiveness, and socio-economic factors, all of which have been shown to impact local transmission patterns [32].

The finding that children aged 5–15 years were more likely to test positive for malaria is consistent with established patterns of age-dependent malaria risk in endemic areas. This relationship has been documented [33] previously and it is thought that children between 5 and 14 generally have higher infection rates (prevalence) because they have historically been low priority in malaria control activities (ITNs) and they often have irregular sleeping patterns which means they are often not under a net. School-age children are among the least likely age groups to sleep under insecticide-treated nets because parents have less control over their sleeping habits as they grow older [34]. Similarly, other studies [35, 36] have highlighted low ITN utilization among school children in Kenya despite high household net ownership. Another study [37] further demonstrated that sleeping arrangements and difficulty in hanging nets significantly affected ITN use among children aged 5–14 years in western Kenya.

The predictive value of vector density and HBR measurements, obtainable through relatively simple sampling methods, can be leveraged to optimize surveillance in resource-limited settings. This approach is supported by recent work demonstrating successful implementation of simplified entomological monitoring for routine surveillance [38]. However, while community-based entomological surveillance presents opportunities for cost-effective expansion of data collection [39, 40], recent evaluations in western Kenya highlight challenges with accuracy of mosquito identification by community collectors, requiring continued investment in training and quality assurance [39]. Given the WHO's emphasis on strengthened surveillance systems and the recent global increase in malaria cases [41], integrating entomological indices into routine malaria monitoring can enhance early warning systems and guide targeted interventions. Recent experiences from Côte d'Ivoire demonstrate how entomological monitoring data can effectively drive decision-making for appropriate vector control interventions, including stratified ITN distribution strategies [42]. The observed spatial heterogeneity supports targeted interventions, an approach shown to be cost-effective [43].

A number of limitations with important ramifications for ongoing malaria surveillance were identified in this study. The four-month study duration may not have captured seasonal transmission variations, and broader geographical coverage would strengthen regional applicability. Reliance on health facility records may underestimate true disease burden, a common limitation in surveillance studies [44]. Entomological surveillance relied on indoor CDC light traps, and human biting rates were estimated indirectly rather than through human landing catches, potentially underestimating outdoor transmission and true biting rates. Also the households selected for entomological surveillance may not have corresponded exactly to the population represented by the health-facility records, so the two data sources described overlapping but not identical populations. In addition, molecular identification and sporozoite testing were performed on a subset of mosquitoes, and the relatively small number of infected mosquitoes may have reduced the precision of EIR estimates, contributing to the lack of association between EIR and malaria test positivity. Finally, the malaria test positivity rate is not equivalent to parasite prevalence and should be interpreted as a facility-based indicator of transmission rather than a population prevalence estimate.

Future studies should consider expanding to diverse eco-epidemiological zones as demonstrated in recent regional assessments [28, 29], which enable more generalizable conclusions for intervention planning. Similar studies should incorporate year-round surveillance across diverse transmission settings, combine routine health facility data with community-based surveillance, include both indoor and outdoor mosquito sampling methods, and evaluate additional environmental, behavioural, and intervention-related factors that may influence the relationship between entomological and epidemiological indicators. Such studies would provide more comprehensive evidence to support integrated malaria surveillance and guide targeted vector control strategies.

## Conclusion

Vector density and human biting rate may serve as easily measurable predictors of malaria transmission intensity that can optimize surveillance strategies in high-endemicity settings. The observed spatial heterogeneity suggest that malaria control could be improved by targeting vector control efforts in high-transmission areas identified through health facility surveillance. These findings support the integration of simplified entomological surveillance into routine malaria programs as a means of tracking transmission patterns and guiding control efforts.

## Data Availability

Yes. I generated research data in this study.
